# Supplementary material for: Calmodulin shuttling mediates cytonuclear signaling to trigger experience-dependent transcription and memory
Source: Nat Commun. 2018 Jun 22;9:2451. doi: 10.1038/s41467-018-04705-8 (PMC6015085; doi:10.1038/s41467-018-04705-8)
Supplement: Supplementary file 1 — Supplementary Information [file 41467_2018_4705_MOESM1_ESM.pdf]

Calmodulin shuttling mediates cytonuclear signaling to trigger experience-dependent transcription and memory

Samuel M. Cohen[1,2,*], Benjamin Suutari[2,3,*], Xingzhi He[1,*], Yang Wang[1], Sandrine Sanchez[2], Natasha N. Tirko[2], Nataniel J. Mandelberg[2], Caitlin Mullins[2], Guangjun Zhou[1], Hongyan Shi[1], Ilona Kats[2], Alejandro Salah[2], Richard W. Tsien[2,3,†], Huan Ma[1,†]

[1]Department of Physiology and the First Affiliated Hospital, Mental Health Center, Institute of Neuroscience, Key Laboratory of Medical Neurobiology of Ministry of Health of China, Zhejiang University School of Medicine, Hangzhou 310058, China.

[2]NYU Neuroscience Institute and Department of Neuroscience and Physiology, NYU Langone Medical Center, New York, NY 10016, USA.

[3]Center for Neural Science, New York University, New York, NY 10003, USA.

*These authors contributed equally to this work

[†]Correspondence: richard.tsien@nyumc.org (R.W.T.), mah@zju.edu.cn (H.M.)

Supplementary Figures

Statistical analysis was performed with one-way ANOVA followed by Holm-Sidak posthoc test unless otherwise noted. $^*p \leq 0.05$, $^{**}p \leq 0.01$, $^{***}p \leq 0.001$. Error bars represent SEM unless otherwise noted.

Supplementary Figure 1

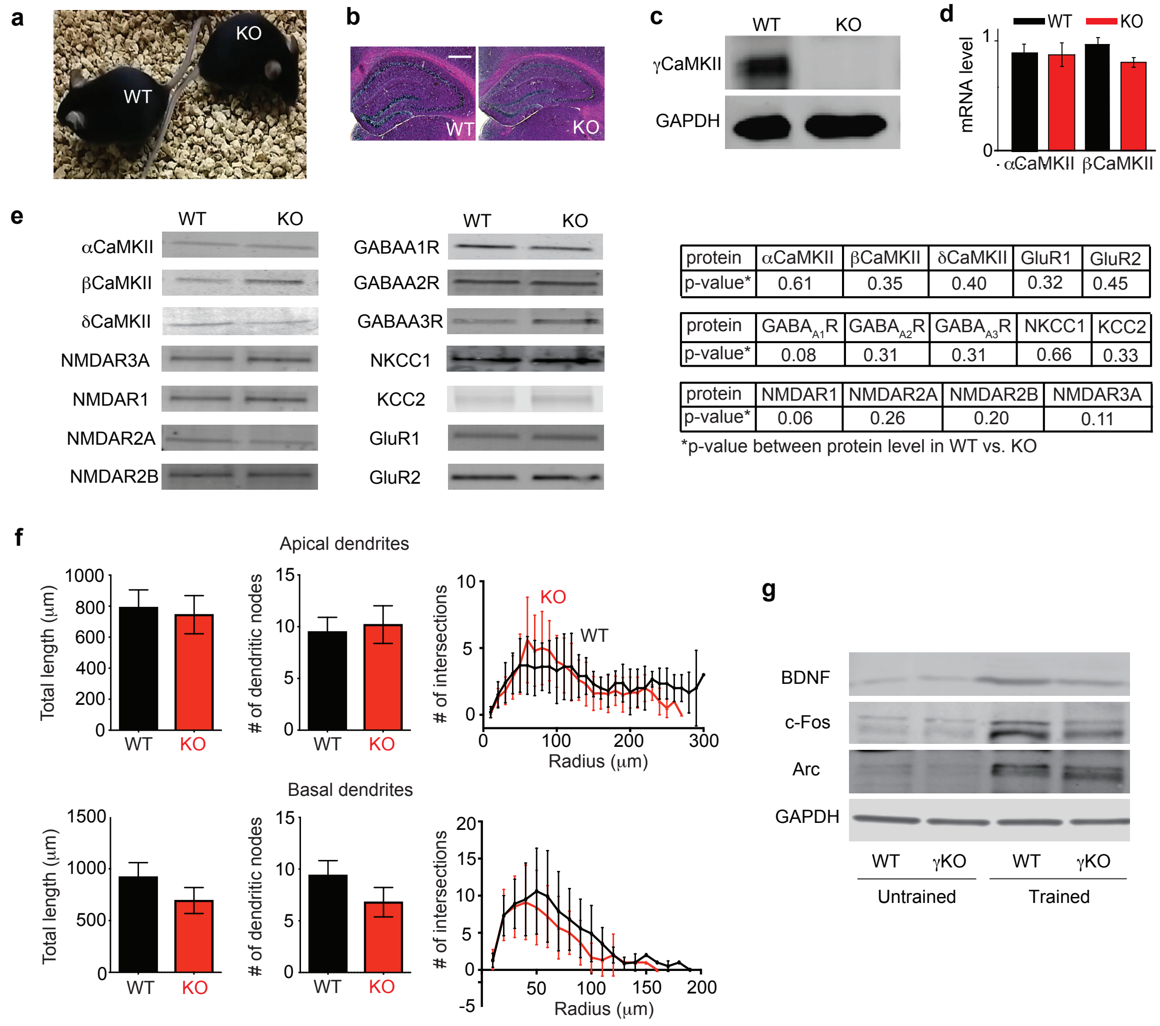

protein	αCaMKII	βCaMKII	δCaMKII	GluR1	GluR2
p-value*	0.61	0.35	0.40	0.32	0.45

protein	$GABA_{A1}R$	$GABA_{A2}R$	$GABA_{A3}R$	NKCC1	KCC2
p-value*	0.08	0.31	0.31	0.66	0.33

protein	NMDAR1	NMDAR2A	NMDAR2B	NMDAR3A
p-value*	0.06	0.26	0.20	0.11

*p-value between protein level in WT vs. KO

Supplementary Fig. 1. Characterization of γCaMKII KO mice

(a) Top, γCaMKII KO mice show no obvious morphological defects compared to their WT littermates at the same age (3 mo). (b) HE staining in the hippocampus of WT and γCaMKII KO mice did not reveal any apparent abnormalities at the macroscopic level. Scale bar, 500 μm (c) The specificity of a custom antibody against γCaMKII (amino acids 441–460) *in vivo* was shown with western blot in brain tissues from WT and γCaMKII KO and confirms complete deletion of γCaMKII in γCaMKII KO mice. (d) No

difference in the expression of αCaMKII or βCaMKII in the brain between WT and γCaMKII KO mice ($p>0.05$, as determined by Student's t test). (e) Knockout of γCaMKII also spared levels of δCaMKII and other critical neuronal activity-related proteins. Left, raw western blot data. Right, statistical analysis. (f) Golgi staining analysis showed no difference between WT and KO mice in total length, number of dendritic nodes, or number of intersections (Sholl analysis) in apical dendrites (top row) or basal dendrites (bottom row) of pyramidal neurons. ($p>0.05$ for all comparisons, N=3 mice for each condition, with 3-5 neurons analyzed per mouse. Error bars in plot on right, SEM). (g) Western blot showing raw data on levels of BDNF, c-Fos, and Arc in the hippocampus of WT and γCaMKII knockout mice, before and after 3 days of training in the MWM test. Data correspond to the plot shown in Fig. 1b.

Supplementary Figure 2

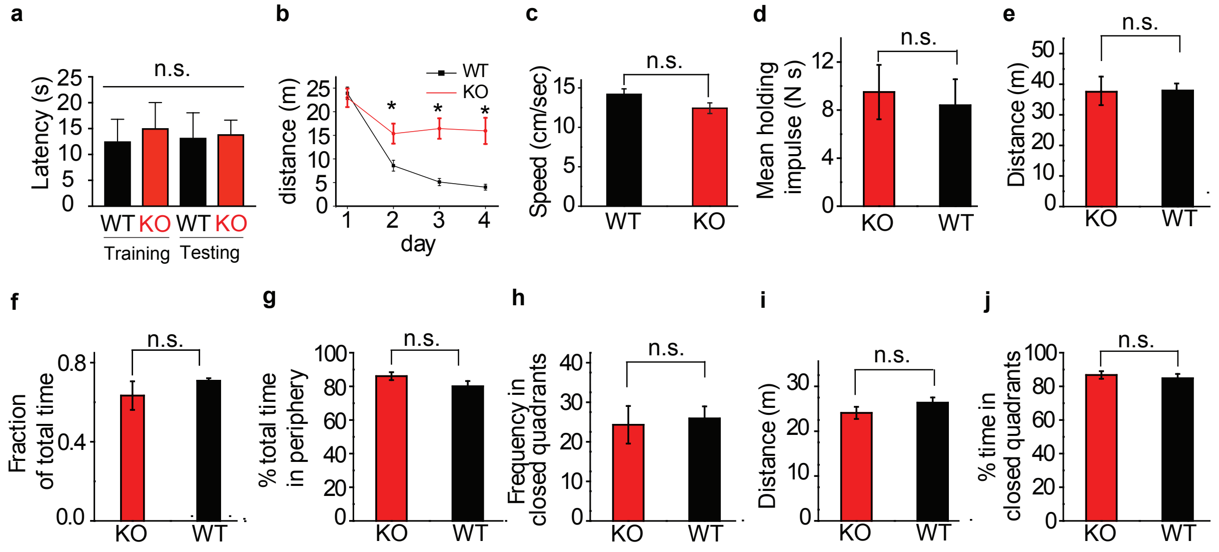

Supplementary Fig. 2. Normal anxiety, locomotor activity and musculoskeletal function in γCaMKII KO mice.

(a) Inhibitory avoidance test, control experiment. Latency to enter dark compartment during training trial and during a testing trial 24 hr later, with mild shock omitted. (b) Spatial memory acquisition and retrieval were tested with the MWM. Mean distances traveled to reach a hidden platform were plotted against the training day (4 trials per day) for WT (black, n=21 for the 1st three days and 12 for the 4th day) and γCaMKII KO (red, n=19 for the 1st three days and 10 for the 4th day) mice. (c) Swimming ability was assessed in MWM, and there was no difference for swimming speed between γCaMKII KO mice and their WT littermates. (d) Muscle function was tested in four limb-hanging test, in which a wire grid was used to measure the ability of mice to exhibit sustained limb tension to oppose their weight (n=10 for both genotypes, p>0.7, one-way ANOVA followed by Holm-Sidak posthoc test). (e-g) Open field behavior. Monitoring of locomotor activity and anxiety in a novel, relatively large environment over 10 min.

γCaMKII KO mice and their WT littermates showed a similar distance moved (e), time spent moving (f), and time in the periphery of the novel environment (g). (h-j) Elevated zero maze behavior. Mice were exposed to an elevated, annular platform with two closed and two open quadrants for 8 min. No significant difference between γCaMKII KO mice and their WT littermates for frequency of entry into closed quadrants (h), distance moved (i) and % time spent in closed quadrants (j).

Supplementary Figure 3

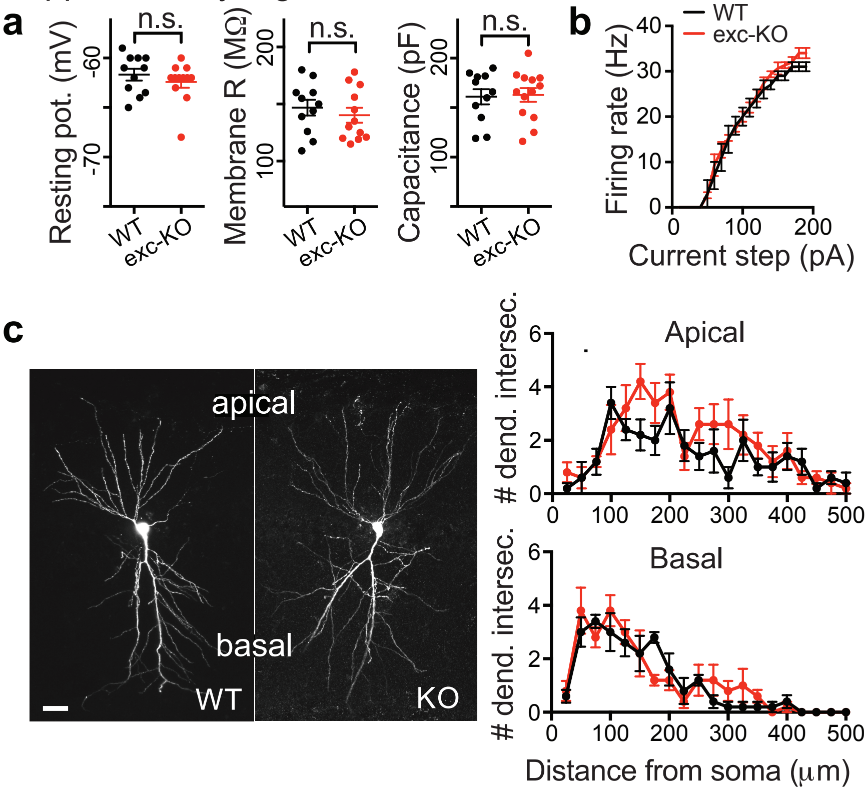

Supplementary Fig. 3. Characterization of excitatory neurons from γCaMKII exc-KO mice

(a) No significant differences detected between CA1 pyramidal cells of WT and γCaMKII exc-KO mice with regard to resting potential ($p>0.3$), input resistance ($p>0.4$) or capacitance ($p>0.8$). (b) No significant difference between WT and γCaMKII exc-KO with respect to firing frequency vs applied current (F-I) curves ($p>0.3$). (c) Sholl analysis of CA1 pyramidal cell morphology revealed no differences in dendritic branching between WT and γCaMKII exc-KO mice for either basal ($p>0.6$) or apical ($p>0.2$) arbors (WT: n=5 cells from 3 mice; exc-KO: n=5 cells from 3 mice; 2-way ANOVA; scale bar, 50 μm).

Supplementary Figure 4

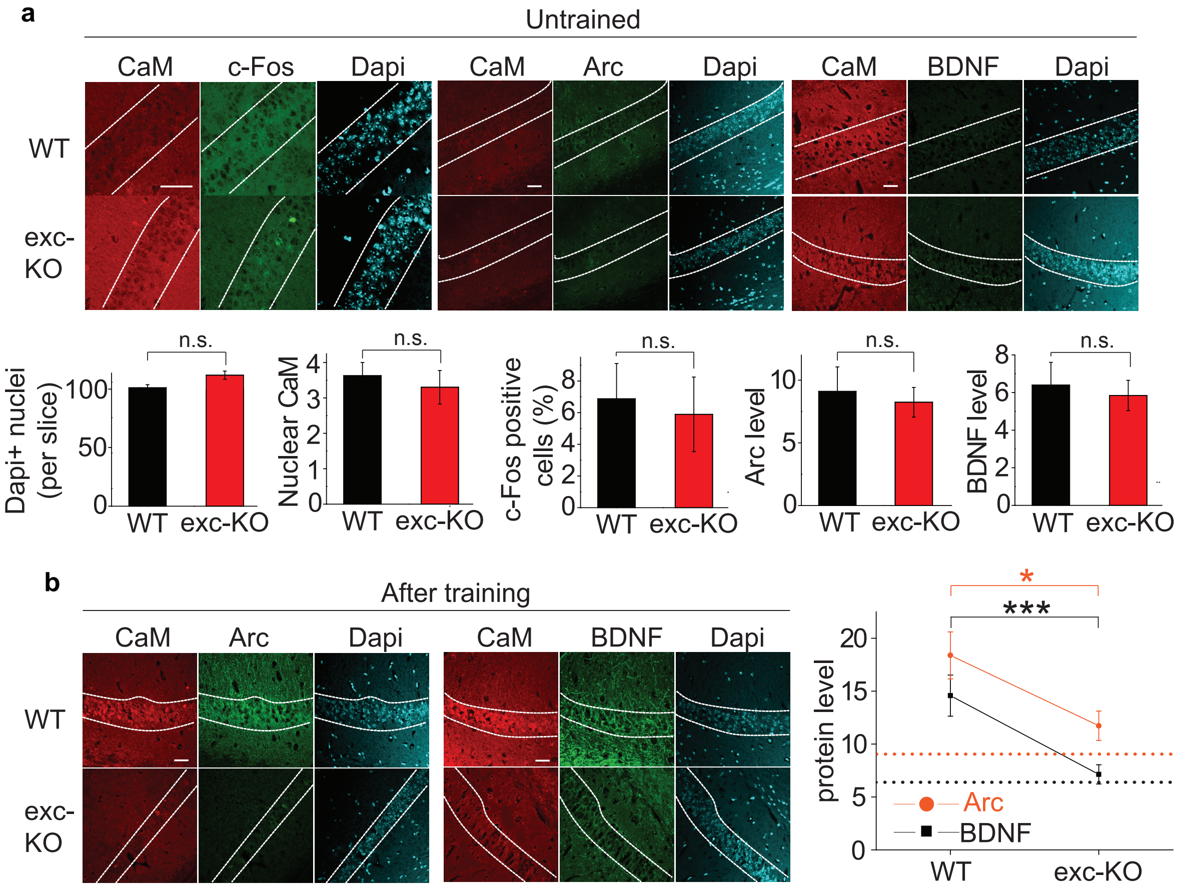

Supplementary Fig. 4. Activity-dependent CaM translocation depends on the shuttle protein γCaMKII and drives CREB-dependent gene expression.

(a) Representative images showing DAPI, nuclear CaM level, and the expression of c-Fos, BDNF and Arc in the CA1 region of WT mice before MWM training, which are all similar in comparisons between γCaMKII KO and WT mice. Quantification is shown in bar graphs. Scale bars, 40 μm. (b) Representative images show increases in nuclear CaM and expression of BDNF and Arc in the CA1 region of WT mice at 1 hr after MWM training, that are absent in γCaMKII exc-KO mice. Scale bar, 10 μm. Quantification of nuclear CaM level and the level of BDNF and Arc in the CA1 region at 1 hr after MWM

training in WT and γCaMKII KO mice (n=5 mice for each group, $p=0.02$ for Arc and $p=0.0009$ for BDNF, one-way ANOVA followed by Holm-Sidak posthoc test).

Supplementary Figure 5

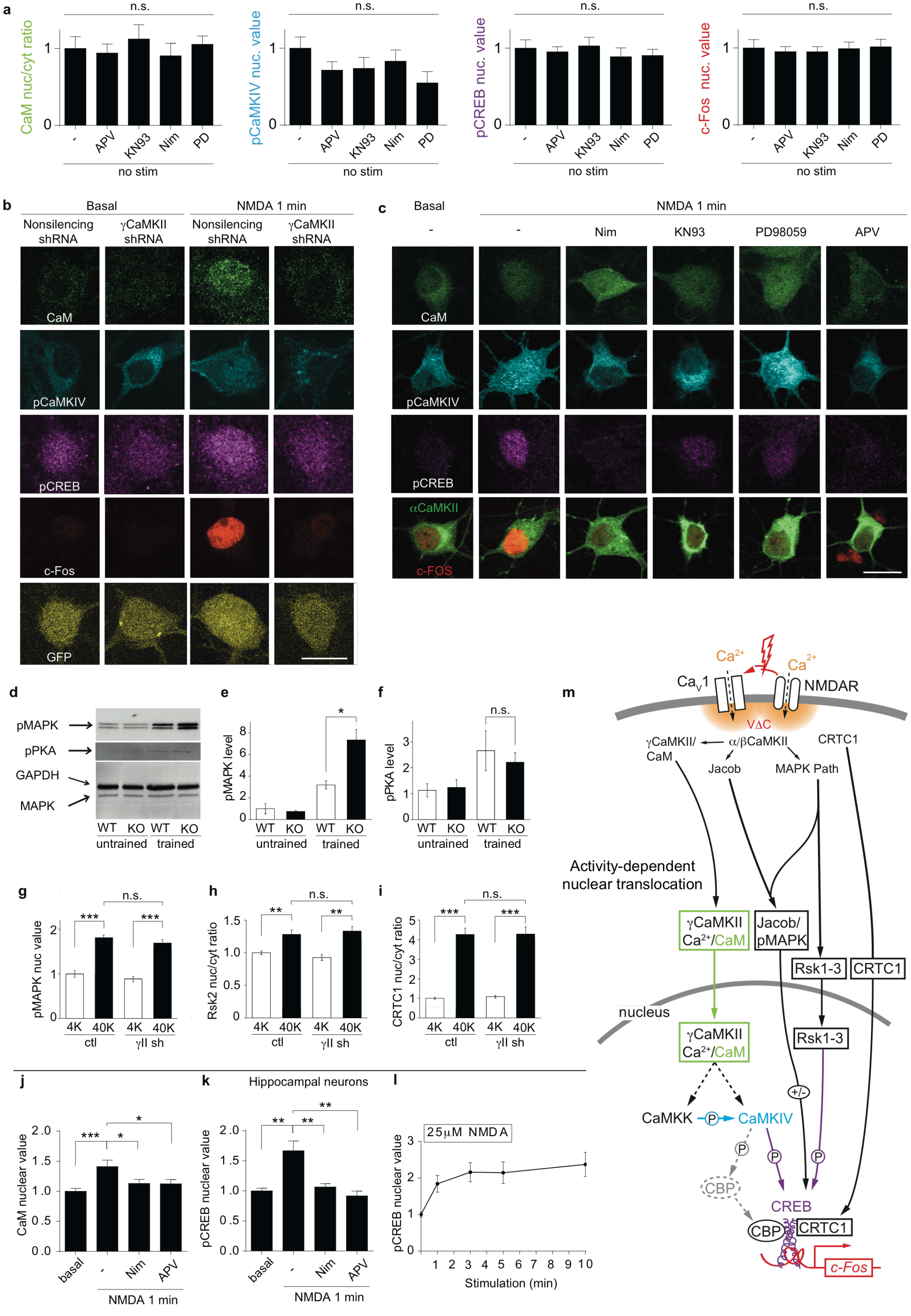

Supplementary Fig. 5. γCaMKII KO is not necessary for activation of PKA or MAPK, or for nuclear translocation of CRTC1 or Rsk2.

(a) CaM nuclear/cytoplasm value and nuclear values of pCaMKIV, pCREB, and c-fos were unaffected by treatment with APV, KN93, Nimodipine, or PD98059 in the absence of NMDA stimulation. (b,c) Representative images of immunostaining for CaM, pCaMKIV, pCREB, and c-Fos with and without NMDA stimulation in cultured excitatory neurons. For quantification (see Fig. 2), the average nuclear pixel value of pCaMKIV, pCREB, and c-Fos, and the nuclear/cytoplasm ratio of CaM, was analyzed using Dapi counterstaining to choose ROIs. (b) Cells transfected with γCaMKII shRNA or a control nonsilencing shRNA construct tagged with GFP (see Fig. 2d-g). Scale bar, 20μm. (c) Cells treated with a variety of pharmacologic inhibitors (see Fig. 2h-k). Bottom row also shows representative αCaMKII staining used to identify excitatory neurons. Scale bar, 20μm. (d-f) *In vivo* tests for confounding crosstalk with other key pathways. After MWM training, the activation of PKA and MAPK was monitored using specific antibodies for phosphorylation at Ser96 of PKA and p44/42 MAPK. The phosphorylation (and, by proxy, activation) of MAPK (e) and PKA (f) was spared in the hippocampus of γCaMKII KO mice after MWM training. (N≥3 mice). (g-i) Cultured cortical neurons expressing a γCaMKII shRNA (denoted γII sh) or a nonsilencing control were depolarized with 40 mM KCl for 10 min and stained with specific antibodies; γCaMKII knockdown did not affect the intensity of phospho-MAPK staining (g), nuc/cyt ratio of Rsk2 (h), or nuc/cyt ratio of CRTC1 (i). (j-l) In cultured hippocampal neurons, stimulation with 25 μM NMDA/ 5 μM Glycine triggered increases in nuclear CaM staining (j) and CREB phosphorylation, within 1 min (k, l). These increases were prevented by nimodipine or APV (j, k). All plots

are the average of two experiments with >25 cells per condition, *$p<0.05$, **$p<0.01$, ***$p<0.001$ (one-way ANOVA followed by Student's *t*-test). (m) Schematic depicting γCaMKII/CaM pathway and representatives of other alternative signaling pathways to the nucleus (NFκB and PKA not shown for sake of simplicity). In addition to CaMK-dependent signaling[19, 27, 44], Rsk2 has an important role in CREB phosphorylation[16], CRTC1 is a critical coactivator of CREB[13], and Jacob translocation modulates CREB activation in synergy with pMAPK signaling[14]. Based on control experiments (d-l), none of the effects of γCaMKII knockdown can be explained by perturbations of these pathways.

Supplementary Figure 6

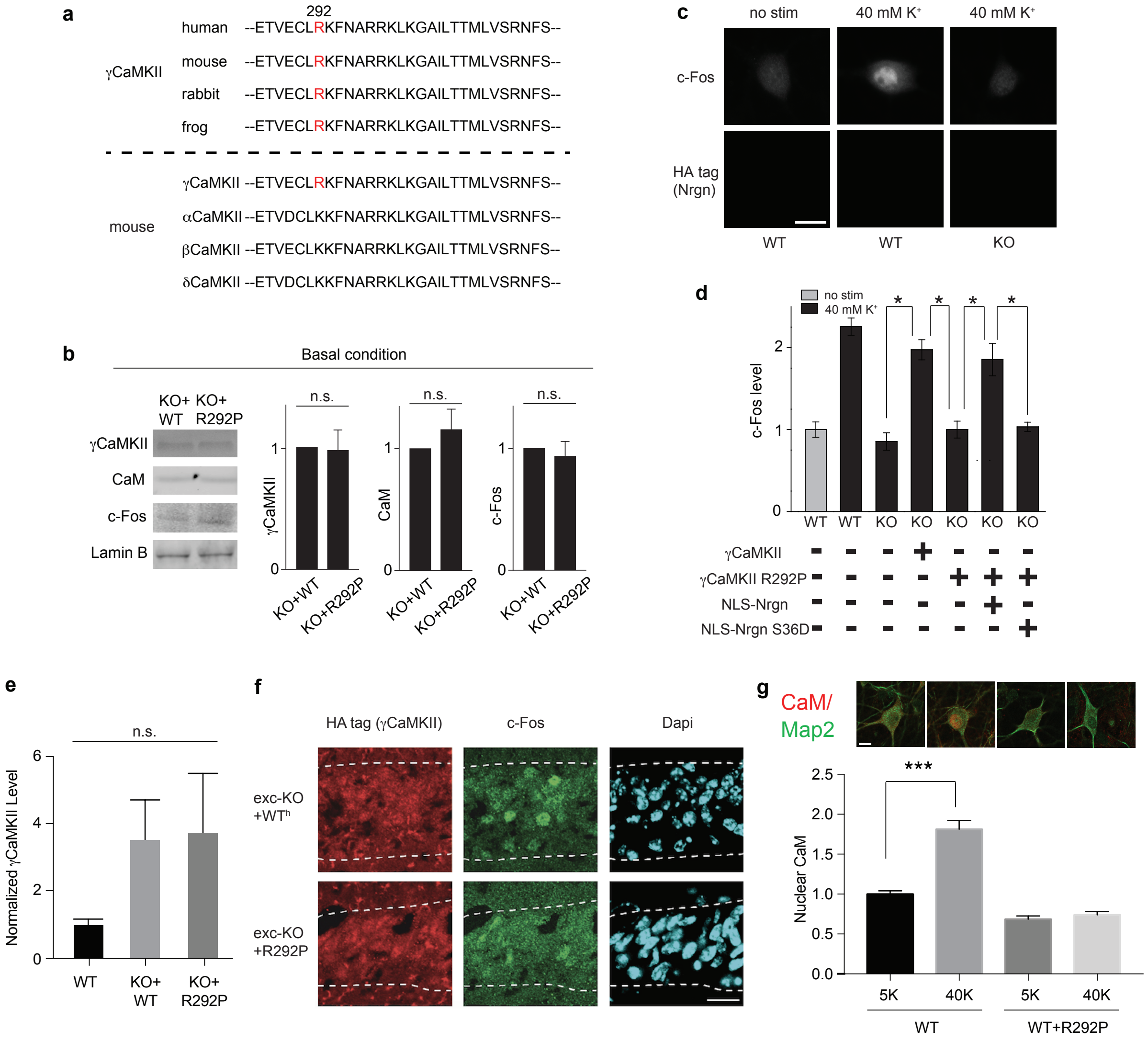

Supplementary Fig. 6. γCaMKII R292P is unable to trap CaM and shuttle CaM into the nucleus to regulate gene expression.

(a) The arginine residue R292 of γCaMKII is highly conserved across different species and differs from the lysine found in other CaMKII isoforms. (b) Western blot data (raw data on left, quantification on right) showing levels of γCaMKII, CaM, and c-Fos in cultured hippocampal and cortical neurons from γCaMKII KO mice transfected with γCaMKII WT or γCaMKII R292P, in the absence of stimulation. (c-d) Data

complementary to results shown in Fig. 3k. Cultured hippocampal and cortical neurons from WT or γCaMKII KO mice were left resting (no stim) or stimulated with 40 mM KCl for 1 hr. (c) c-Fos was expressed in WT but not γCaMKII KO mice with 40K stimulation. HA staining is part of a negative control to confirm the specificity of staining for HA-tagged Nrgn (see also Fig. 3k). Scale bar, 10 μm. (d) Collected results corresponding to immunostaining data illustrated in panel b and Fig. 3k. Note that under basal conditions, overexpressing NLS-Nrgn or NLS-Nrgn S36D (visualized with HA tag staining in Fig. 3k) did not affect c-Fos levels in the presence of γCaMKII R292P. (N=3 cultures for each group, $p<0.002$, two-way ANOVA followed by Holm-Sidak posthoc test). (e) Normalized level of γCaMKII, assessed by western blot, in the hippocampus of WT mice or γCaMKII KO mice infected with γCaMKII WT, γCaMKII R292P. Normalization is with respect to a GAPDH loading calibration, as well as γCaMKII WT as 1.0. (f) Staining of HA (γCaMKII) and c-Fos in CA1 of γCaMKII exc-KO mice injected with HA-γCaMKII or HA-γCaMKII R292P; illustrates deficit in c-Fos expression despite arrival of HA-γCaMKII in nucleus. Slices are from mice used in Fig 4a, f that were sacrificed 1 hr post-training. Scale bar, 20 μm. (g) Immunostaining for CaM, in the presence or absence of 40K stimulation, in cultured hippocampal neurons taken from WT mice and transfected with either γCaMKII R292P or with an empty vector. Nuclear CaM normalized to basal condition for control neurons. ***, $p<0.001$. Scale bar, 10 μm.

Supplementary Figure 7

Related to Figure 3

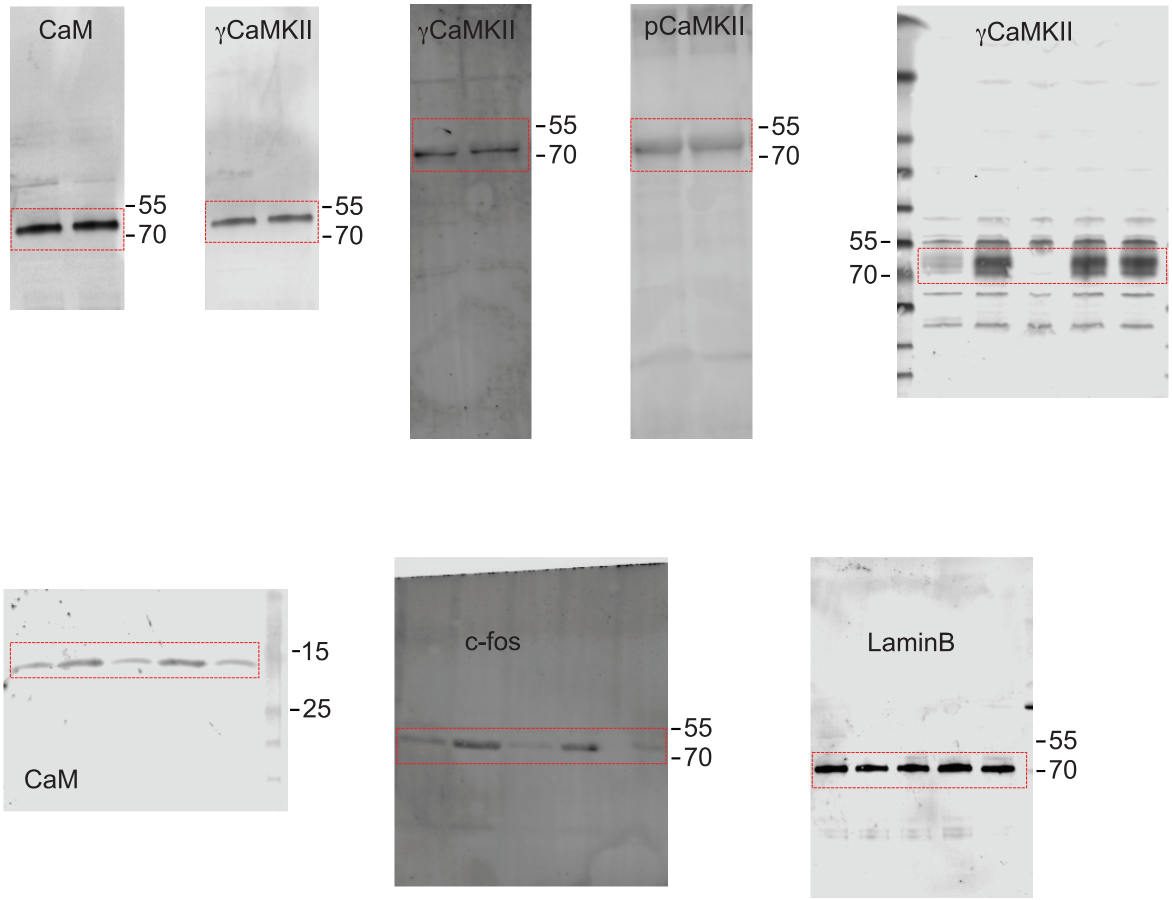

Supplementary Fig. 7. Uncropped blots and gels data for data in Figure 3.

Supplementary Figure 8

Related to Supplementary Figure 1

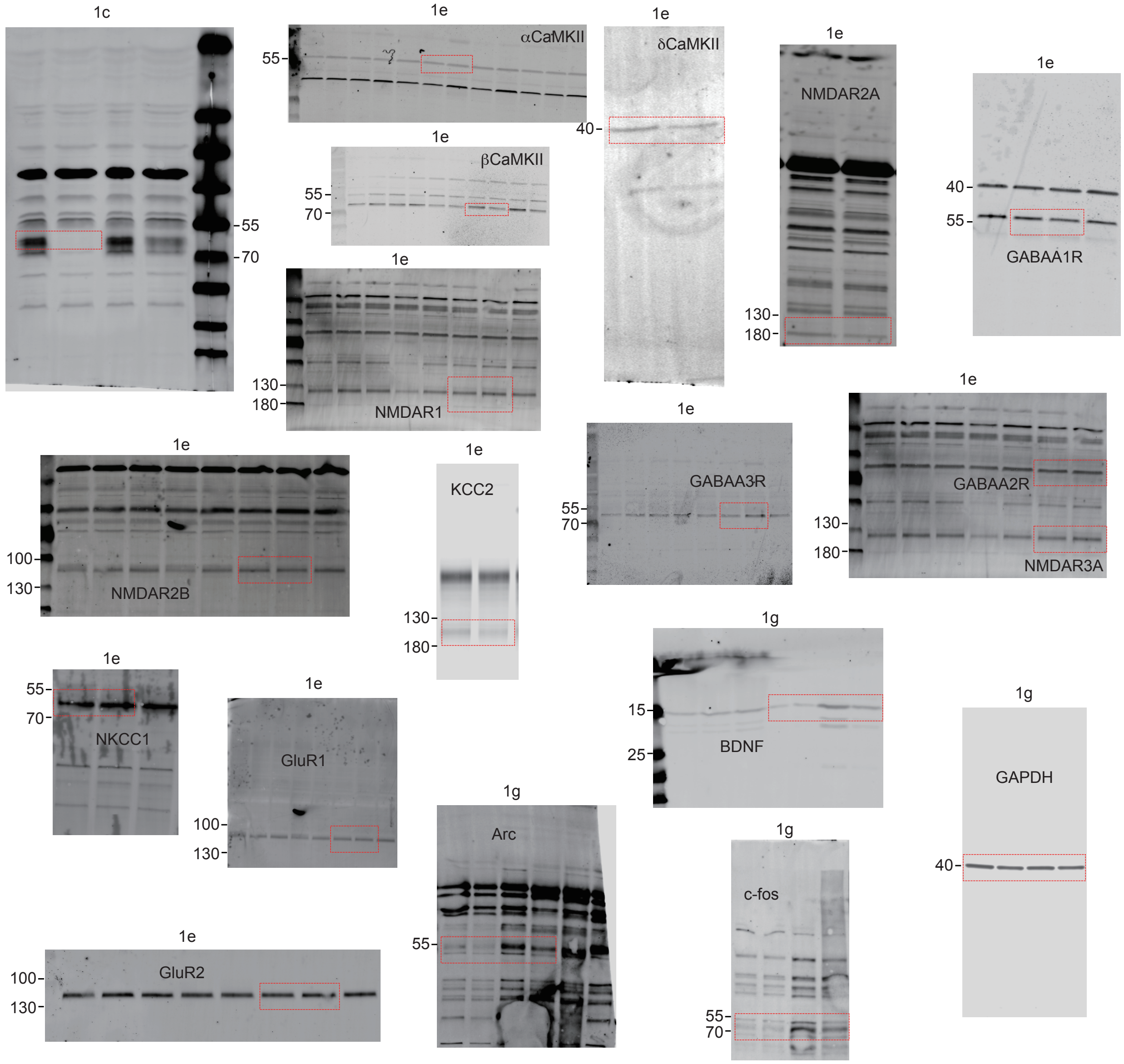

Supplementary Fig. 8. Uncropped blots and gels for data in Supplementary Figure 1

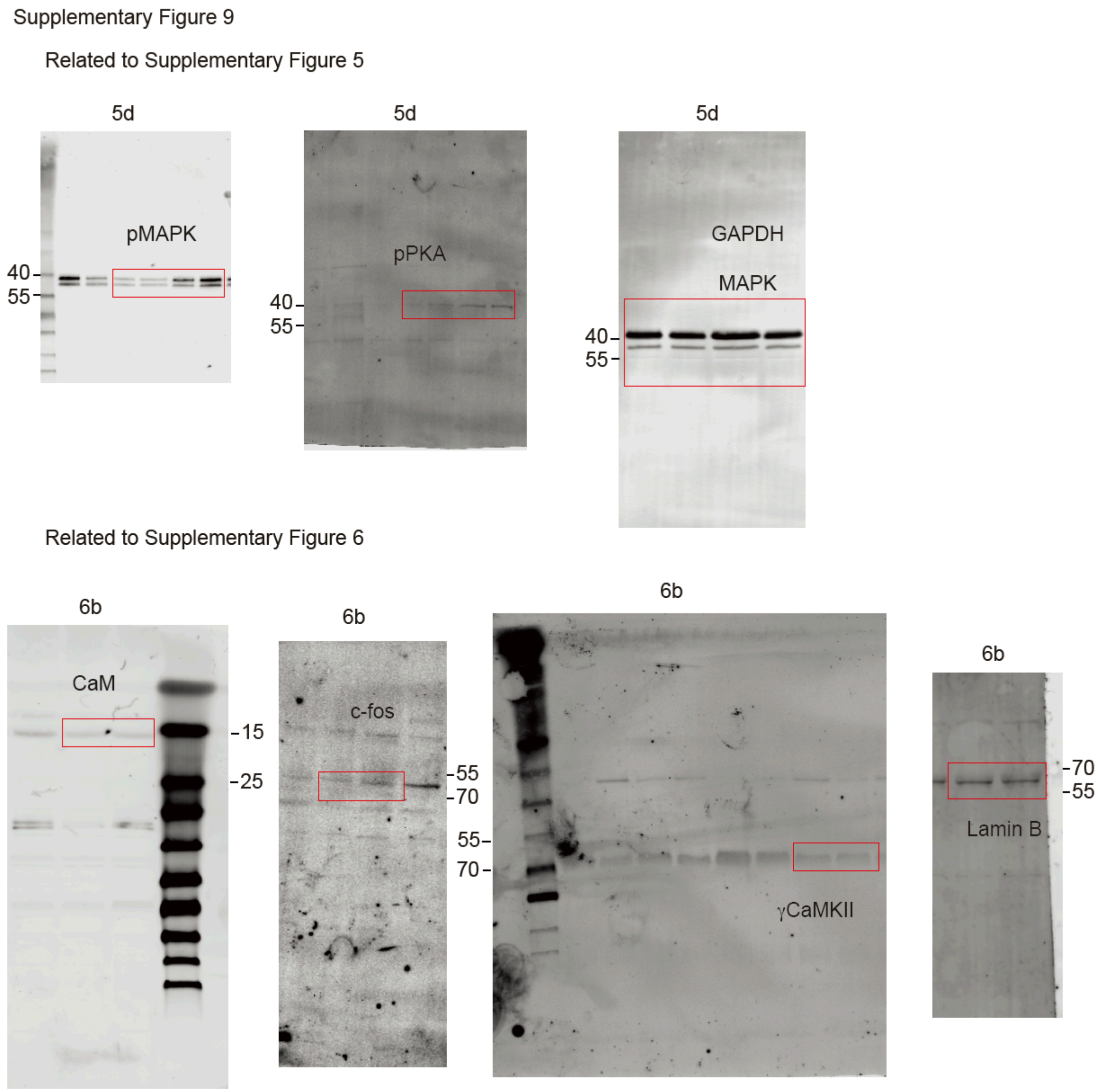

Supplementary Fig. 9. Uncropped blots and gels for data in Supplementary Figures 5 and 6.
